# SteE Enhances the Virulence of *Salmonella* Pullorum in Chickens by Regulating the Inflammation Response

**DOI:** 10.3389/fvets.2022.926505

**Published:** 2022-07-14

**Authors:** Zhike Liu, Li Wang, Yan Yu, Anatoliy Fotin, Qiuxia Wang, Pei Gao, Yanhong Zhang, Tetiana Fotina, Jinyou Ma

**Affiliations:** ^1^College of Animal Science and Veterinary Medicine, Henan Institute of Science and Technology, Xinxiang, China; ^2^Faculty of Veterinary Medicine, Sumy National Agrarian University, Sumy, Ukraine

**Keywords:** *Salmonella* Pullorum, *steE*, HD-11, cytokine, colonization, virulence

## Abstract

*Salmonella enterica* serovar Pullorum (*S*. Pullorum) is a host-specific pathogen, which causes acute gastroenteritis with high mortality in poultry. However, the association between *steE*, encoded by type III secretion system 2, and *Salmonella* virulence is not well-understood. To elucidate the functions of *steE* in *S*. Pullorum, Δ*steE* strain was constructed using the λ-Red recombination technology. Compared to that in the wild-type, the deletion of *steE* in *S*. Pullorum reduced bacterial invasion, proliferation, and late apoptosis in the infected HD-11 cells. In addition, we analyzed the mRNA expression levels of effector genes and cytokines by qRT-PCR. *SteE* was associated with the regulation of various effector genes and inflammatory cytokines in HD-11 cells during *S*. Pullorum infection. The wild-type effector *steE* promoted the expression of anti-inflammatory cytokines (IL-4 and IL-10) and reduced that of pro-inflammatory cytokines (IL-1β, IL-6, and IL-12) compared to that in the Δ*steE*-infected HD-11 cells and chicken spleens. Results from the chicken infection model showed that the deletion of *steE* resulted in significantly decreased colonization and long-term survival of the bacteria and alleviated pathological lesions compared to those in the wild-type. Further, *steE* increased the virulence of *S*. Pullorum in chickens by regulating the expression of inflammatory cytokines. Our findings provide insights into the persistent infection and autoimmunity associated with *steE* in *S*. Pullorum.

## Introduction

*Salmonella* is an intracellular pathogen causing great harm to human and livestock health. It has complex and diverse antigenicity and serotypes. Among the prevalent serotypes, *S*. Pullorum causes septic diseases in poultry, with chronic or acute infection in adult chickens, and reduces their survival rate after infection. Although clinical signs are not obvious, the egg-laying capacity and meat production are seriously affected (1, 2). In developed countries, the spread of the disease is controlled; however, it is still a persistent disease in the poultry industry in several developing countries including Brazil, China, and India (3, 4).

After invasion, *Salmonella* survives and proliferates in a *Salmonella*-containing vacuole (SCV) inside host cell (5). Effector proteins from *Salmonella* pathogenicity islands 1 and 2 (SPI-1 and SPI-2)-encoded type III secretion systems (T3SSs) are induced in nutrient poor environments. Subsequently, the secreted effectors (*sseF, sspH2, ssaT, hilA*, and *spiC*) necessary for regulating immune cell activity are translocated into the cytosol of infected host cells (6, 7). *Salmonella* also induces an anti-inflammatory response, but the underlying mechanisms unclear. Several *Salmonella* T3SS effector genes (*sseL, gtgA, gogA, gogB*, and *avrA*) can specifically target and suppress the NF-κB pathway during infection. This plays an important role in inhibiting inflammation, regulating apoptosis, and promoting cell proliferation and chronic infection (8–10). A new effector gene of the *Salmonella* T3SS, *steE* is encoded within the Gifsy-1 prophage, which helps *Salmonella* survive and replicate in HD-11 cells. The phage is critical for the evolution of host-specificity and regulation of host innate immunity in *Salmonella*. The effector protein SteE promotes the transition of granulomatous macrophages to M2 polarized macrophages, while persistent *Salmonella* infection overcomes host restriction (11). However, the effects of *steE*, changes associated with cytokine expression in avian HD-11 cells and their contribution toward *Salmonella* virulence are incompletely known.

In the present study, the roles of *S*. Pullorum *steE* in the intracellular replication, host immunity, and virulence were analyzed using the Δ*steE* strain and the HD-11 cells and chickens as infection models.

## Materials and Methods

### Cells, Plasmids, and Primers

Avian HD-11 cells were cultured in Dulbecco's Modified Eagle Medium (DMEM) (Hyclone, UT, USA) with 10% fetal bovine serum (FBS; Invigentech, CA, USA) and 1% penicillin-streptomycin (Solarbio, Beijing, China). Cells were incubated at 37°C in an incubator with 5% CO_2_. The pKD4, pCP20, and pKD46 plasmids were kindly provided by Professor Ya-wei Sun (Henan Institute of Science and Technology, China) for the λ-Red recombination technology. Luria-Bertani (LB) media was supplemented with kanamycin (Kan, 50 μg/mL) for *Salmonella* culture as required. Antibiotic-free media was used for 24 h before infection and transfection. The primers used in this study are described in Table 1.

**Table 1:** The primers used in this study.

**Primer**	**Sequences (5^**′**^-3^**′**^) Forward primer/reverse primer**	**Size (bp)**
*steE*-cat-F	CGGGTGGCGATTTTAACGCCAGTGCGACGTTAGTCGTGGATTACCAgtgtaggctggagctgcttc	1,140
*steE*-cat-R	AACATTACGCCTCCGATCAAATGCCCGGCAGTTTGAAAAATACGGTcatatgaatatcctccttag	
CX1/ CX2	ATTCAGGGAACCACCACCAT/ACGCCAATCGCAAAACCACT	1089/683/2169*
*β-actin*	TATTGCTGCGCTCGTTGTTGAC/GATACCTCTTTTGCTCTGGGCTTC	181
IL-1β	ATGTCGTGTGTGATGAGCGGC/AGGCGGTAGAAGATGAAGCGG	107
IL-4	AGTGAATGACATCCAGGGAGAGG/CTGACGCATGTTGAGGAAGAGAC	172
IL-6	AAATCCCTCCTCGCCAATCTG/CCTCACGGTCTTCTCCATAAACG	105
TNF-α	TGTCTGCTCCTAGTGGCTTTCC/TTGGCATAGGCTGTCCTGAGT	165
IL-10	CGCTGTCACCGCTTCTTCAC/GGCTCACTTCCTCCTCCTCATC	99
iNOS	TGGTAACAGCGGAAGGAGACA/TTCCAGGACCTCCAGGATGTT	110
TGF-β1	TCCAATGTAGCCACCACCAA/ACAGGGACAAGACGCAAACC	121
IL-12	TGCCTTACTTTCATTACTTTCCTTTG/TTTAGCTGGTGTCTCATCGTTCC	109
*gmK*	CTTCTTCGCTATCTTGCCCG/ACCATTGAGCAAGTGCTGGC	172
*spiC*	CCATCCGCTGTGAGCTGTAT/CCGAAGGTAATAGCCGATCC	199
*sefC*	GCCAATTGACATGGCAAGCA/TGAGCAATCACCCCACCAGA	172
*sspH2*	TCCACTCCCTGAACTCGCTT/AAAGGTCAGAACGCTGGCTC	197
*sseJ*	CTTATCGGCGTGTTCCTGTG/GCAGAGGCGCTCGAATGTAT	156
*ssaT*	TTGAGCGGCATTGAGAGGAA/AGGCAGAGTGGAGAACGCTT	145
*sseF*	TTTGTTCAGGCGTAAGCAGC/TTCCGTCAGCGGCAAGTAAT	138
*pipB*	ACCCGTTGACATCCTCCAGA/CACGCGGTATACTGGAATGG	171
*sseC*	AGCCTCCTCTGCCATCTCATT/TTGGCGAGGAAGTGGTTGAG	158
*sipA*	CTTTCGGATGAAGCGTTGGT/CGACTACGCATCAAACGGAG	127
*hilA*	ACGGACAGGGCTATCGGTTT/TCTTCGTAATGGTCACCGGC	201
*sseG*	GGAGACGGCTTTAGCAATCG/GCGGATGTCGCCTGTCTTAT	138
*ssaV*	GCGATAATGATACCGCCGAT/GATTTGCGTGCCGGAGTTAT	173
*sifB*	GGCGGCTTTTCTTTCCTGTT/GCTTGTTCCCTGAGCGGTTA	143
*sipC*	GCAACGGCACTGGAAGACAT/GTCACGACTAAAGCGAATGAGG	104
*steA*	GTATCGGTAATGGCACGCTG/GTCAGTCTTCATCAGCGCGA	182
*prgH*	CGCAAACTGCACATAGCGTC/CAGGCGTTACCTTATTCCCG	147
*steB*	AAGTTTAGCGGGCAGCACAC/CTTCCGACATCCGCAATCAC	126
*steE*	ACGGTGAAATGCTGGAGGTC/CATCGCGAAAGCTGCTGTC	114

Lowercase letters: Kan^R^ cassette amplification; *A 1089 bp fragment was amplified from S. Pullorum, and 683 bp and 2,169 bp fragments were amplified separately from S. Pullorum ΔsteE and ΔsteE::Kan, respectively.

### Construction of the Δ*steE* Strain

*S*. Pullorum (CVCC 530; China Veterinary Culture Collection, Beijing, China) was used as the reference strain and cultured in LB medium at 37°C for 12 h. The Δ*steE* strain was generated using the λ-Red recombination technology (12). Briefly, with the pKD4 plasmid as a template, the kanamycin resistance cassette (Kan^R^) was amplified *via* PCR using the specific primers *steE*-cat-F/R, including 46 bp homology extensions from the sequence of *steE* (GenBank: LK931482.1). Purified PCR products from the Kan^R^ cassette were transformed into the wild-type (WT) strain carrying the pKD46 plasmid via electroporation. The resulting *S*. Pullorum Δ*steE*::Kan strain was identified via PCR analysis using the primer pair CX1/CX2. The Kan^R^ cassette gene in *S*. Pullorum Δ*steE*::Kan was excised via introducing the pCP20 FLP expression plasmid using electroporation. The *S*. Pullorum Δ*steE* strain was verified *via* PCR analysis.

### *Salmonella* Growth Curve Assay

The WT and Δ*steE* strains were inoculated into LB broth and grown at 37 °C with shaking at 180 rpm for 12 h. The next day, overnight cultures of *Salmonella* were added to 20 mL of LB media (1:100 dilution). Subsequently, the optical density (OD) of the mixtures was adjusted after dilution to reach 0.01 OD/mL, and the samples were cultured at 37 °C with shaking at 180 rpm. The WT and Δ*steE* strains were cultured in LB liquid medium for 16 h and the OD_600_ of the bacterial cultures was recorded each hour using the BioDrop spectrophotometer (BioDrop, Cambridge, England) to evaluate the growth curve of the bacteria.

### Infection of HD-11 Cells With WT and Δ*steE* Strains

HD-11 cells were used for the cell infection assay as described previously (13). Cells were plated at 2 × 10^5^ cells per well on a six-well-plate and cultured overnight until 80–90% confluency was obtained. For the bacterial adhesion assay, HD-11 cells were infected with overnight cultures of the WT and Δ*steE* strains at a multiplicity of infection (MOI) of 10:1. Subsequently, the inoculated six-well-plate was centrifuged at 500 × *g* for 10 min to promote the interaction of the cells with *Salmonella*. After 1 h incubation at 37 °C, the cells were lysed with 1 mL of 0.1% of Triton X-100 (Sangon Biotech, Shanghai, China) for 10 min. The cell lysates of the WT and Δ*steE* strains were serially diluted 10-fold using phosphate-buffered saline (PBS) and the dilutions were spread on LB agar before incubation at 37 °C for 12–16 h to analyze the adhesive ability of bacteria. For the bacterial invasion assay, at 1 h after infection, HD-11 cells were washed three times with PBS and incubated for another 1 h in DMEM with 10% FBS and 100 μg/mL gentamicin (Solarbio, Beijing, China) to kill extracellular bacteria. HD-11 cells were subsequently lysed with 1 mL of 0.1% Triton X-100 for 10 min and plated to calculate the number of colonies. For the bacterial proliferation assay, the infected host cells were washed with PBS and incubated in DMEM supplemented with 10% FBS and 10 μg/mL gentamicin; this step was set as the 0 h time point. At 0, 3, 6, 9, 15, and 20 h time-points, HD-11 cells from each well were lysed with 1 mL of 0.1% Triton X-100 for 10 min. The cell lysates at 10-fold serial dilutions were plated for colony-forming unit (CFU) analysis. The number of intracellular bacteria was calculated and presented as the fold-change at the indicated time points compared to the initial numbers present at 0 h.

For identification of effector genes and cytokines induced by the WT and Δ*steE* strains, infected host cells were incubated in DMEM with 10% FBS and 10 μg/mL gentamicin for 3, 4, 8, and 16 h. Infected cells were washed with PBS, and total RNA was prepared using the TRIzol reagent (Invitrogen, Carlsbad, USA). cDNA synthesis using 1 μg total RNA was performed using the PrimeScript RT reagent kit with gDNA Eraser (Takara, Dalian, China), and the extracted RNA was stored at −80 °C until qRT-PCR analysis. HD-11 cells stimulated with lipopolysaccharide (LPS, 10 μg/mL) (Sigma, CA, USA) were used as the positive reference group for mRNA expression analysis.

### Cellular Apoptosis Assay

HD-11 cells (2 × 10^5^ per well) were plated on a six-well-plate and incubated for 16–18 h at 37 °C. Briefly, WT and Δ*steE* strains from overnight culture in LB media were washed with sterile PBS. Subsequently, the bacterial suspensions were diluted to reach ~ 1 × 10^8^ CFU/mL. The WT and Δ*steE* strains were used to infect HD-11 cells at an MOI of 10:1, and the mix was cultured at 37 °C in an incubator at 5% CO_2_. After a 3 h incubation, apoptosis was evaluated using an Annexin V-FITC/PI apoptosis detection kit (Beyotime Biotechnology, Shanghai, China). Approximately 5 μL Annexin V-FITC and 10 μL PI working solutions were added to the cells in each group and mixed gently. Subsequently, the cells were incubated within an ice box at room temperature (20–25 °C) for 20 min in the dark. Cells undergoing apoptosis in different groups were analyzed using the BD LSR Fortessa™ flow cytometer (BD Biosciences, CA, USA) within 1 h. The apoptosis ratios were calculated using the FlowJo 10.6.2 software (Tree Star, OR, USA).

### Chicken Infection Assay

Animal experiments were reviewed and approved by the Laboratory Animal Care and Ethics Committee of Henan Institute of Science and Technology (Permit Number: 2020HIST016), in accordance with international law. For bacterial colonization and pathogenicity assays, 30 Jinghong laying hens (2-day-old) were randomly assigned to three groups, with each group containing 10 chickens. The chickens in the experimental groups were orally infected with the WT or Δ*steE* strain (1 × 10^9^ CFU/chicken) using 100 μL PBS. The chickens in the control group were treated orally with 100 μL PBS. The experimental procedure and inoculation dose used was in accordance with the method described by Yin et al. (14). At 3 days after infection, the liver, spleen, and bursa tissues were collected from each chicken. After weighing, the tissues of five chickens from each group were homogenized mechanically. Appropriate dilutions were plated onto xylose lysine deoxycholate (XLD, Hopebio Bio-Technology, Qingdao, China) agar and incubated at 37 °C for 14–16 h. Bacteria were counted and noted as log_10_ CFUs/g. The livers and spleens were fixed with 10% formalin for 48 h, embedded in paraffin, and cut into 4 μm sections with a paraffin slicer (Leica, Wetzlar, Germany) for histological analysis. The hematoxylin and eosin-stained sections were visualized under a light microscope.

For *in vivo* competition assays, 12 Jinghong laying hens (3-day-old) were randomly assigned to two groups (6 chickens in each group). The chickens were infected orally with 2 × 10^8^ CFUs of a 1:1 mixture of WT and Δ*steE*::Kan strains prepared in 100 μL PBS (6), and the chickens were sampled from each group at 3 days post-infection (dpi). Liver, spleen, and bursa tissues were collected from each chicken as described above. After weighing, tissues from six chickens in each group were homogenized mechanically. Appropriate dilutions were plated on XLD/XLD (Kan^R^) agar and incubated at 37 °C for 14–16 h to calculate the total number of bacteria. The competitive index (CI) was defined using the following formula: the ratio of Δ*steE*/WT strains in the output divided by the ratio of Δ*steE*/WT strains in the input (15).

For survival assays, 30 Jinghong laying hens (3-day-old) were randomly assigned to three groups (10 chickens per group). The chickens in the experimental groups were orally infected with 1 × 10^9^ CFU of the WT or Δ*steE* strain in 100 μL PBS. Additionally, chickens in the control group were infected orally with 100 μL of PBS (5). Death was monitored daily for 20 dpi, and the survival curves were analyzed to evaluate the differences in virulence between the two strains.

### Changes of Inflammatory Cytokines in the Spleen of Chickens After Infection With WT and Δ*steE* Strains

Thirty Jinghong laying hens (2-day-old) were randomly assigned to three groups, with each group containing 10 chickens. The chickens in the experimental groups were orally infected with the WT or Δ*steE* strains (1 × 10^9^ CFU/chicken) in 100 μL PBS. Ten chickens in the control group were infected orally with 100 μL of PBS. At 1, 3, and 7 days after infection, spleens were collected from each group, total RNA was extracted using TRIzol reagent, and cDNA synthesis with 1 μg total RNA was performed using the PrimeScript RT reagent kit with a gDNA eraser. The extracted cDNA was stored at −80 °C until qRT-PCR analysis.

### qRT-PCR Analysis of Effector Genes and Cytokines

cDNA was prepared from infected HD-11 cells and the spleens of chickens, as described above. qRT-PCR was performed using the QuantStudio 5 system (ABI, USA) with the 2 × SYBR Premix Ex Taq II (Takara, Dalian, China) for analyzing the mRNA expression of effector genes and cytokines (Table 1). β*-actin* of HD-11cells or *gmk* from *S*. Pullorum was used as the internal control (16). The threshold cycle (Ct) values were evaluated to calculate the relative expression levels using the 2^−ΔΔCt^ method (4). All qRT-PCR reactions were analyzed in triplicate for each sample.

### Statistical Analysis

Experimental data are shown as mean ± SEM unless otherwise stated. A one-way ANOVA was performed, and significance was calculated using the GraphPad Prism software (GraphPad Software Inc, California, USA). Significant differences were expressed as ^*^p < 0.05 and ^**^p < 0.01 between two groups.

## Results

### Identification of the Δ*steE* Strain

**Figure 1:**
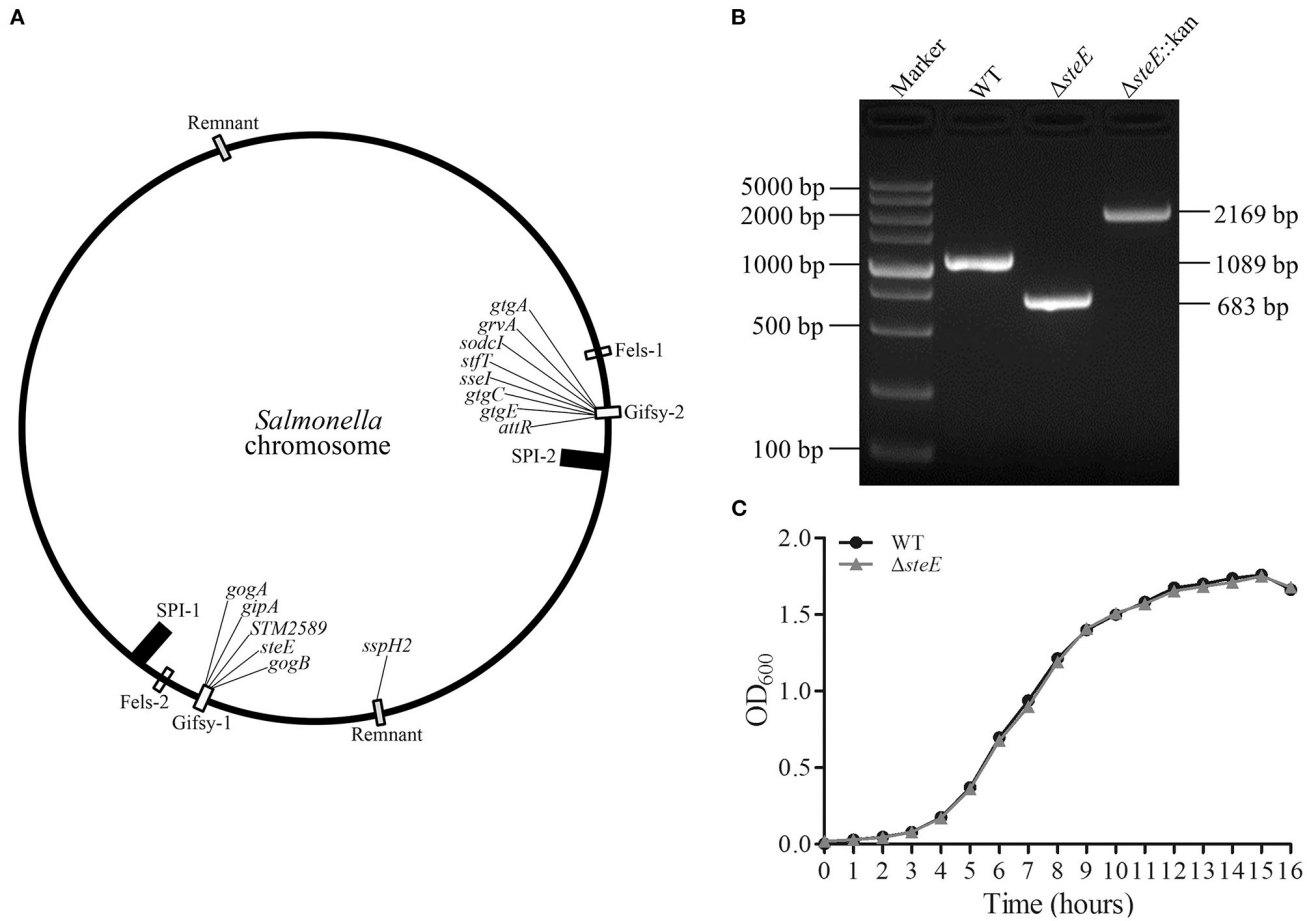
Identification of the Δ*steE* strain. **(A)** The location of *steE* in *Salmonella* chromosome DNA was indicated. The map was depicted from Klumpp and Fuchs (17) and Coombes et al. (18). **(B)** PCR verification of Δ*steE* strain with the CX1/CX2 primer pair. **(C)** Growth curves of the WT and Δ*steE* strains were evaluated by spectrophotometry (OD_600_) in LB media. The experiment was performed in triplicate for each sample.

To elucidate the functions of *steE* in *S*. Pullorum, the Δ*steE* strain was constructed. The gene environment of *steE* is shown in Figure 1A. *steE* is the second open reading frame located in the Gifsy-1 in *Salmonella* chromosomal DNA, indicating horizontal transmission. The construction of Δ*steE* strain was confirmed *via* PCR using the primers CX1/CX2, as shown in Figure 1B. The PCR products from the WT, Δ*steE*, and Δ*steE*:Kan strains had sizes of 1,089, 683, and 2,169 bp, respectively. The PCR results indicated that the Δ*steE* strain was constructed successfully.

To test the effect of *steE* deletion, the growth characteristics of the WT and Δ*steE* strains were evaluated. The growth curves in LB liquid medium were analyzed at the indicated time points, but no significant differences were observed (Figure 1C). The result indicated that *steE* deletion does not significantly affect *S*. Pullorum growth.

### Role of *SteE* in the Adhesive, Invasive, and Proliferative Abilities of *S*. Pullorum in HD-11 Cells

**Figure 2:**
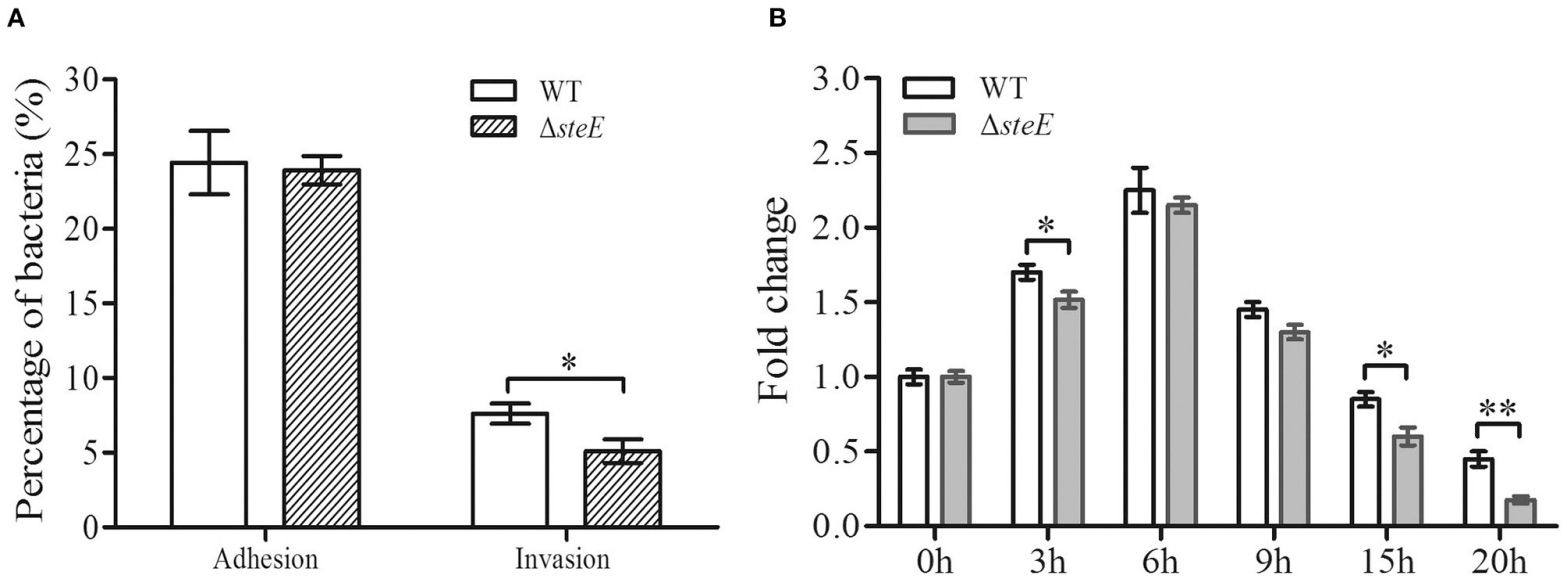
Adhesive, invasive, and proliferative abilities of the WT and Δ*steE* strains in HD-11 cells. **(A)** The percent adhesion and invasion were calculated by comparing bacterial recovery from the primary inoculum at 1 and 2 h post-infection (hpi). **(B)** Proliferation of the WT or Δ*steE* strains within HD-11 cells. It denotes the number of bacteria invading HD-11 cells after a 1 h incubation with 10% FBS and 10 μg/mL gentamicin, which was set as 1 and shown as 0 h time point in the figure. The fold-change is the number of intracellular bacteria at 0, 3, 6, 9, 15, and 20 h/the initial intracellular bacteria present (0 h). *p < 0.05; **p < 0.01.

The adhesive, invasive, and proliferative abilities of the WT and Δ*steE* strains were examined in HD-11 cells. The Δ*steE* strain had no significant effect on *S*. Pullorum adhesion to HD-11 cells (Figure 2A). However, the invasive ability of Δ*steE* strain was significantly reduced compared to that of the WT strain. Additionally, lower proliferation levels were observed with Δ*steE* compared to the WT, and a significant difference was observed at 3, 15, and 20 h in HD-11 cells (Figure 2B). In addition, the intracellular replication of the WT strain showed an increase from 0 to 6 h post-infection (hpi) and subsequently decreased from 6 to 20 hpi. These results indicated that deletion of *steE* reduced the colonization and survival of the bacteria *in vitro*.

### Effector Gene Expression in HD-11 Cells Infected With WT and Δ*steE* Strains

**Figure 3:**
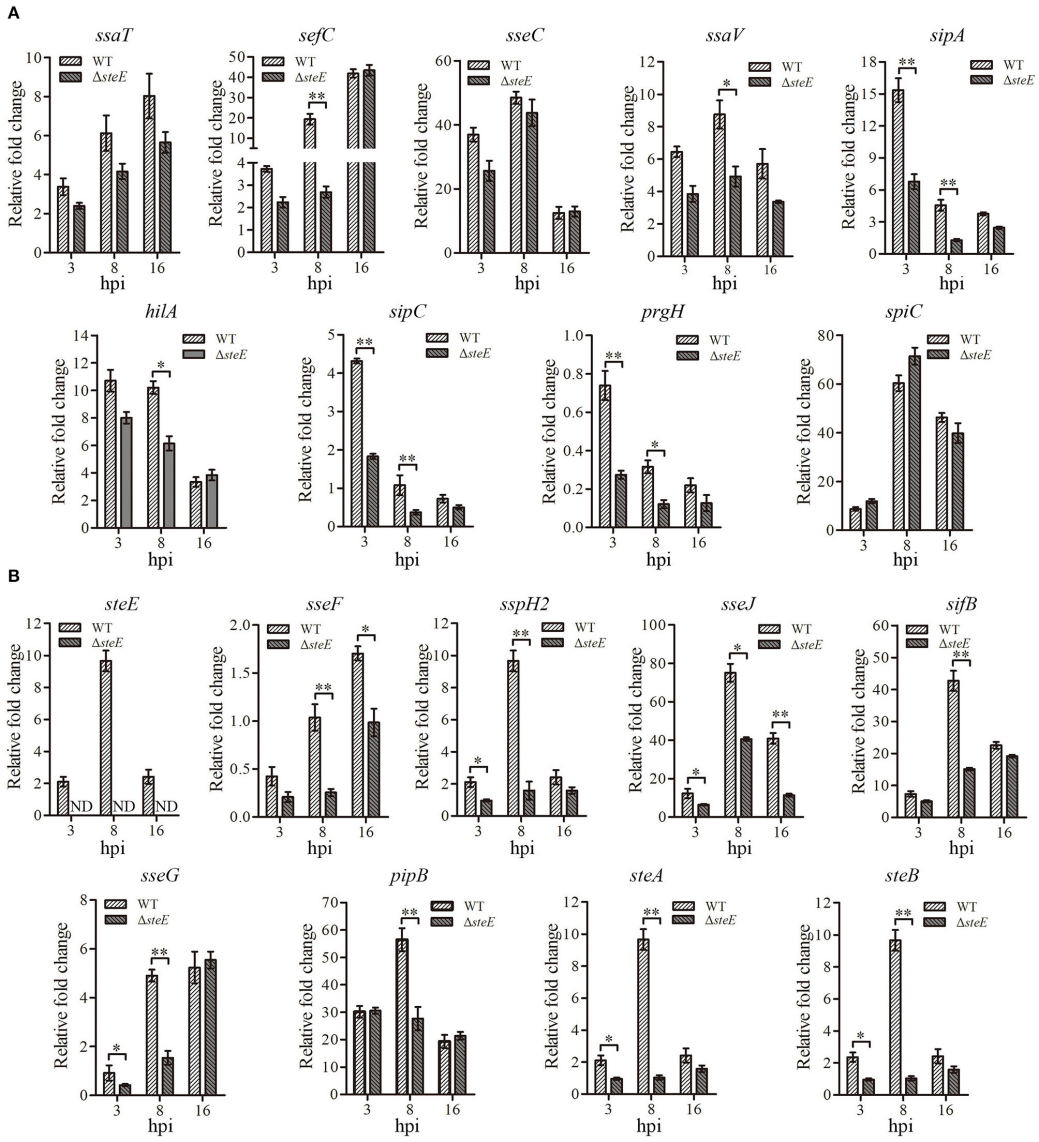
Relative expression levels of effector genes in HD-11 cells infected with the WT and Δ*steE* strains. The expression levels of T3SS1 effector genes *ssaT, sefC, sseC, ssaV, sipA, hilA, sipC, prgH*, and *spiC* **(A)** and the T3SS2 effector genes *steE, sseF, sspH2, sseJ, sseG, sifB, pipB, steA*, and *steB* **(B)** in HD-11 cells infected Δ*steE* strain were evaluated via qRT-PCR at 3, 8, and 16 hpi compared to those with the WT strain at 1.5 hpi. *gmk* was used as an internal control to analyze the relative mRNA levels of effector genes. ND indicates not detected. *p < 0.05; **p < 0.01.

To identify the effects of *steE* deletion on *S*. Pullorum effector genes, mRNA expression levels of the genes were evaluated at 3, 8, and 16 h via qRT-PCR. The transcriptional level of *steE* was not detected in the Δ*steE* strain-infected HD-11 cells, confirming its successful deletion in *S*. Pullorum (Figure 3B). The T3SS1 and T3SS2 effector genes showed differential expression in the WT strain-infected cells. Transcription levels of *sipA, sipC*, and *prgH* were significantly decreased in cells infected with the WT compared to Δ*steE*-infected cells at 3 and 8 hpi, but no significant difference was observed at 16 hpi (Figure 3A). However, the deletion of *steE* significantly reduced the transcriptional levels of *sefC, ssaV*, and *hilA* in HD-11 cells compared to those in the WT at 8 hpi, but the mRNA levels of *ssaT, sseC*, and *spiC* did not significantly differ between the two infected groups at 3, 8, and 16 hpi. The mRNA transcription levels of *sspH2, sseG, steA*, and *steB* were significantly decreased in cells infected with WT compared to those in the Δ*steE-*infected cells at 3 and 8 hpi, and a significant decrease was detected in *sifB* and *pipB* mRNA levels between strains at 8 hpi (Figure 3B). In addition, *sseJ* showed lower levels at three different time points in the Δ*steE* strain-infected HD-11 cells as compared to those in WT, and *sseF* expression was significantly decreased at 8 and 16 hpi (Figure 3B). These results indicated that *steE* reduces the expression of T3SS2 effector genes and few T3SS1 effector genes.

### *SteE* Promotes Apoptosis in HD-11 Cells

**Figure 4:**
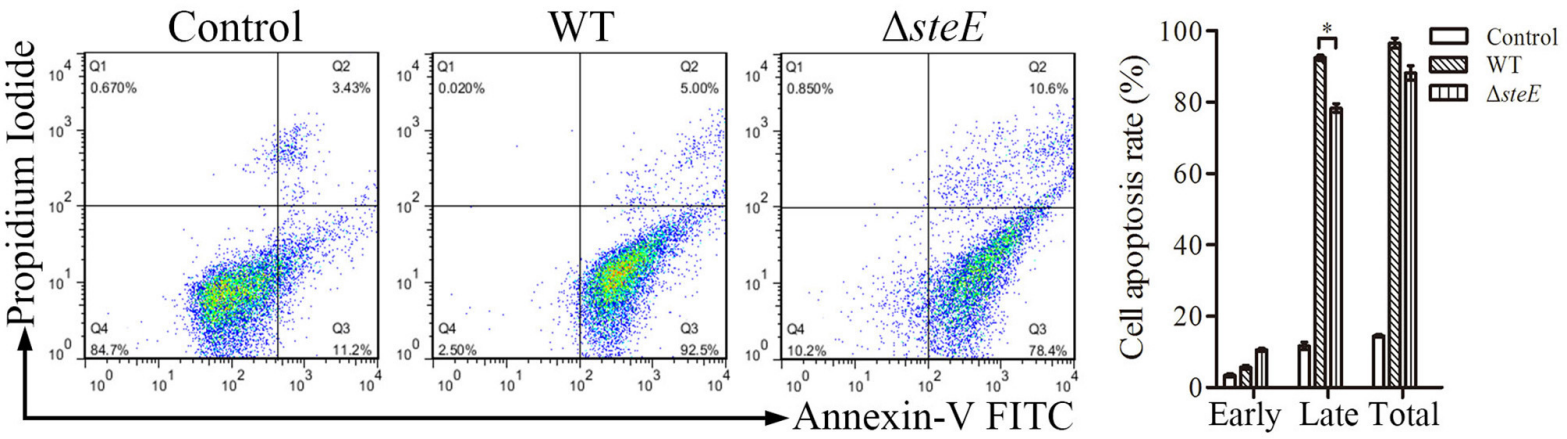
HD-11 cells infected with WT strain promote apoptosis as compared to the Δ*steE* strain. The apoptosis rates were analyzed using flow cytometry. HD-11 cells were collected to analyze total apoptosis (early + late) at 3 hpi. Representative images and the statistical histogram are shown in the left and right panels, respectively. The scatter diagram shows the early (Q2) and late (Q3) apoptosis rates. *p < 0.05.

To assess the effect of *steE* deletion on apoptosis in HD-11 cells, annexin V-FITC/PI analysis was performed. Deletion of *steE* significantly reduced late apoptosis in HD-11 cells as compared to WT strain, although no significant difference was detected for early apoptosis (Figure 4). These results suggest that infection with Δ*steE* significantly reduces late apoptosis in HD-11 cells relative to infection with the WT strain.

### *SteE* Is Required for WT Strain-Induced Cytokine Expression in HD-11 Cells

**Figure 5:**
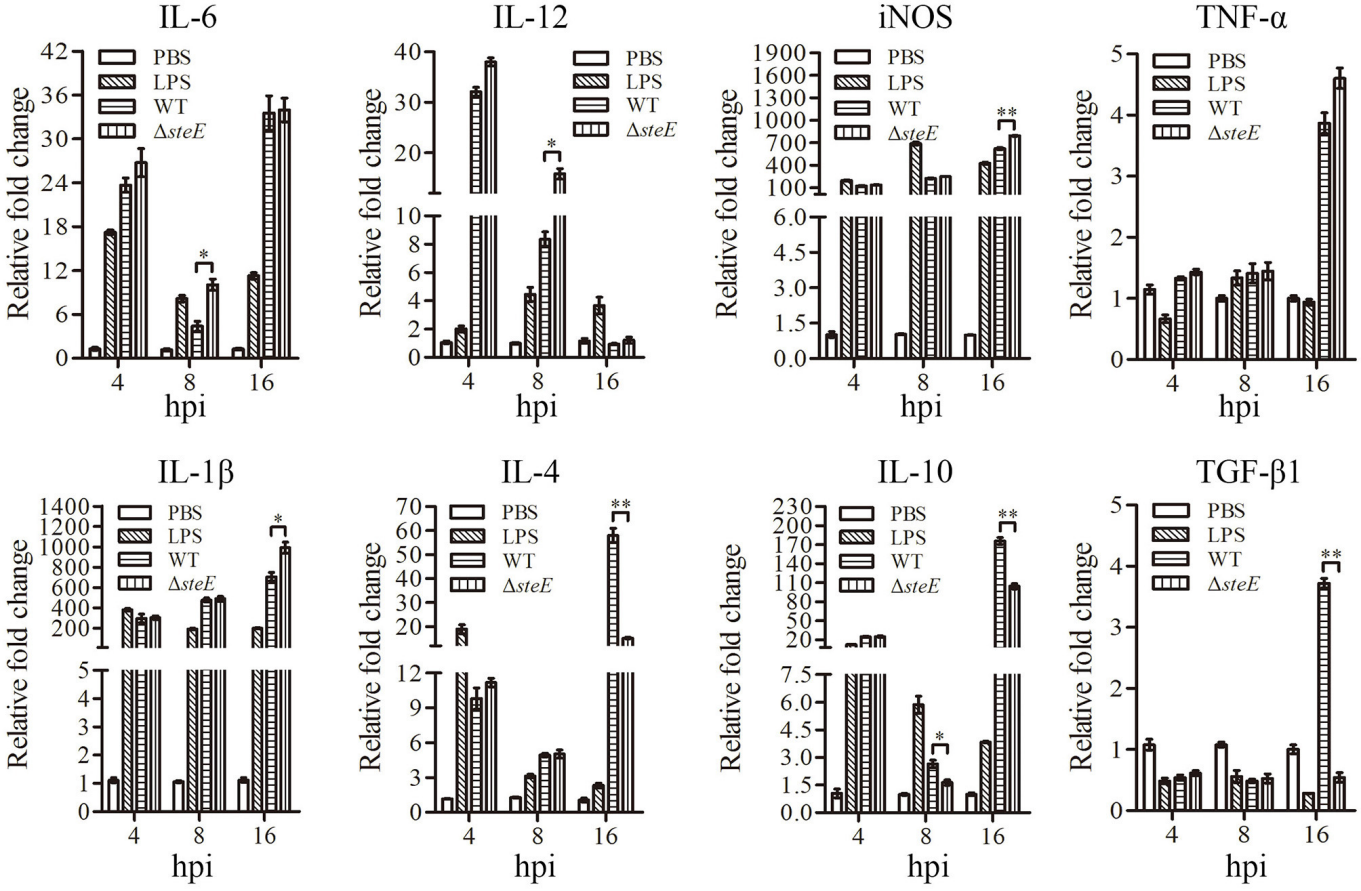
*steE* is integral for *S*. Pullorum-induced cytokine expression in HD-11 cells. Relative expression levels of IL-6, IL-12, iNOS, TNF-α, IL-1β, IL-4, IL-10, and TGF-β1 were evaluated via qRT-PCR at 4, 8, and 16 hpi. The mRNA levels of cytokines were evaluated relative to β*-actin* expression. *p < 0.05; **p < 0.01.

To investigate the effect of *steE* on the immune response *in vitro*, the expression levels of several cytokines were examined using HD-11 cells infected with WT and Δ*steE* strains via qRT-PCR at 4, 8, and 16 hpi. IL-12 expression was strongly increased in Δ*steE* strain-infected HD-11 cells at 8 hpi (Figure 5), but the difference was not significant between the two groups at 4 and 16 hpi. IL-6 expression was higher in the Δ*steE* vs. WT strain-infected group at 8 hpi. However, the expression levels of iNOS and IL-1β were strongly increased in HD-11 cells infected with the Δ*steE* strain compared to those in the WT at 16 hpi. TNF-α expression was similar between the two groups at 4, 8, and 16 hpi. In addition, Δ*steE* strain significantly reduced the expression of the anti-inflammatory cytokine IL-10 in infected HD-11 cells at 8 and 16 hpi; however, IL-4 and TGF-β1 mRNA level differences were significantly lower in HD-11 cells infected with the Δ*steE* strain than in the WT at 16 hpi. These results illustrated the anti-inflammatory effects of *steE* in cells infected with *S*. Pullorum.

### *SteE* Regulates the Expression of Inflammatory Cytokines in Chicken Spleen

**Figure 6:**
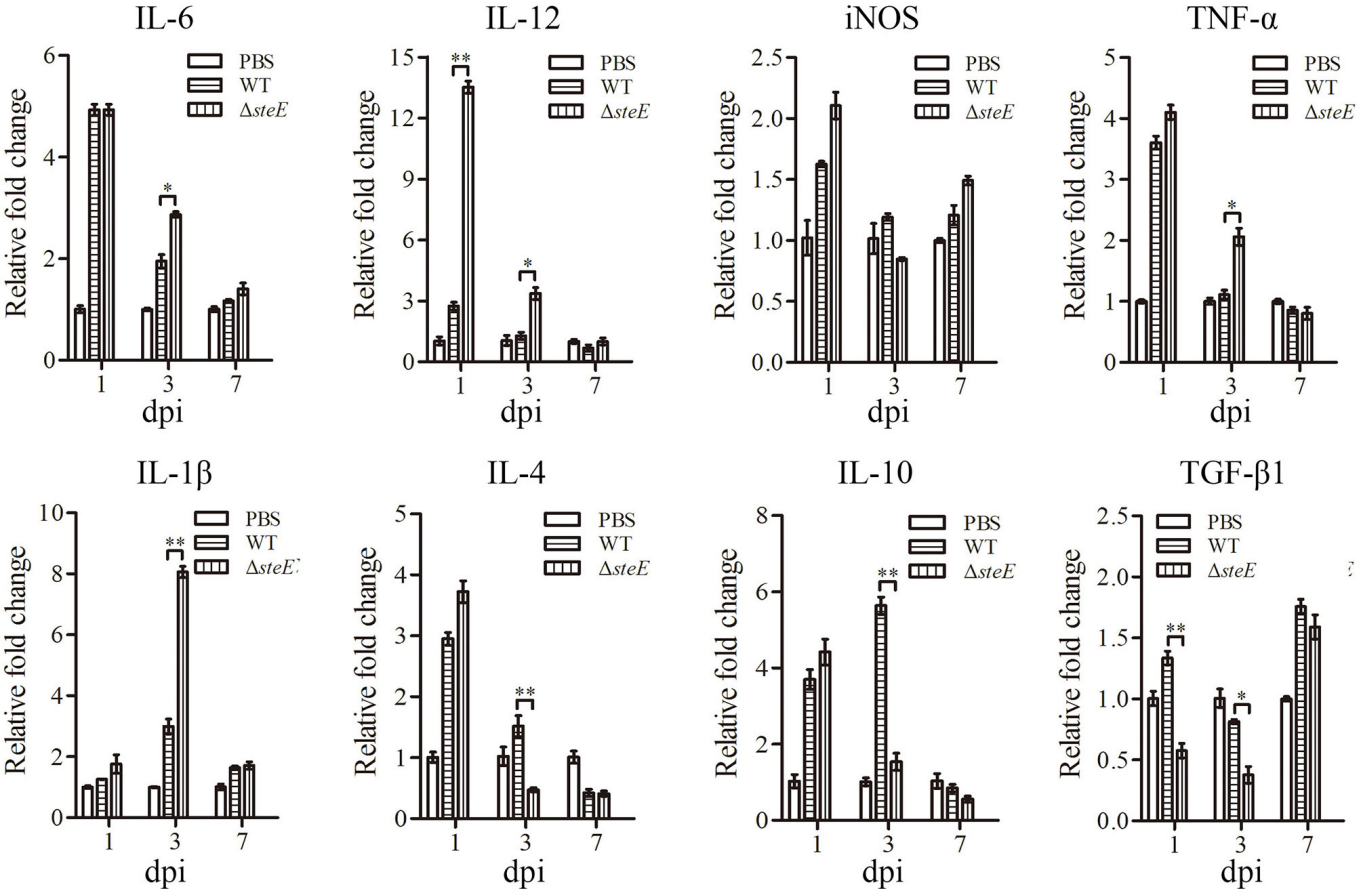
*steE* regulates the expression of inflammatory cytokines in chicken spleen. The expression levels of IL-6, IL-12, iNOS, TNF-α, IL-1β, IL-4, IL-10, and TGF-β1 in chicken spleens infected with WT and Δ*steE* strains were analyzed via qRT-PCR at 1, 3, and 7 dpi. The mRNA levels of cytokines were analyzed relative to β*-actin* expression. *p < 0.05; **p < 0.01.

To test the effect of *steE* on inflammatory responses *in vivo*, cytokines from the spleen of chickens infected with WT and Δ*steE* strains were analyzed via qRT-PCR. IL-12 expression was higher in Δ*steE* vs. WT strain infected group at 1 and 3 dpi, but the difference was not significant between the two groups at 7 dpi (Figure 6). IL-6 expression was significantly higher in the Δ*steE* strain group than that in the WT strain group at 3 dpi, but no difference was detected in iNOS production between the two groups at 1, 3, and 7 dpi. The expression levels of IL-1β and TNF-α were strongly increased in Δ*steE* vs. WT strain group at 3 dpi, but no difference was detected between strains at 1 and 7 dpi. In addition, the anti-inflammatory cytokines IL-4 and IL-10 were significantly lower in the Δ*steE* strain infected group than that in the WT strain group at 3 dpi, though the expression levels of TGF-β1 was decreased significantly at 1 and 3 dpi. The data showed that *steE* was closely associated with the expression of anti-inflammatory and pro-inflammatory cytokines *in vivo*.

### Deletion of *SteE* Reduces Virulence in Chickens

**Figure 7:**
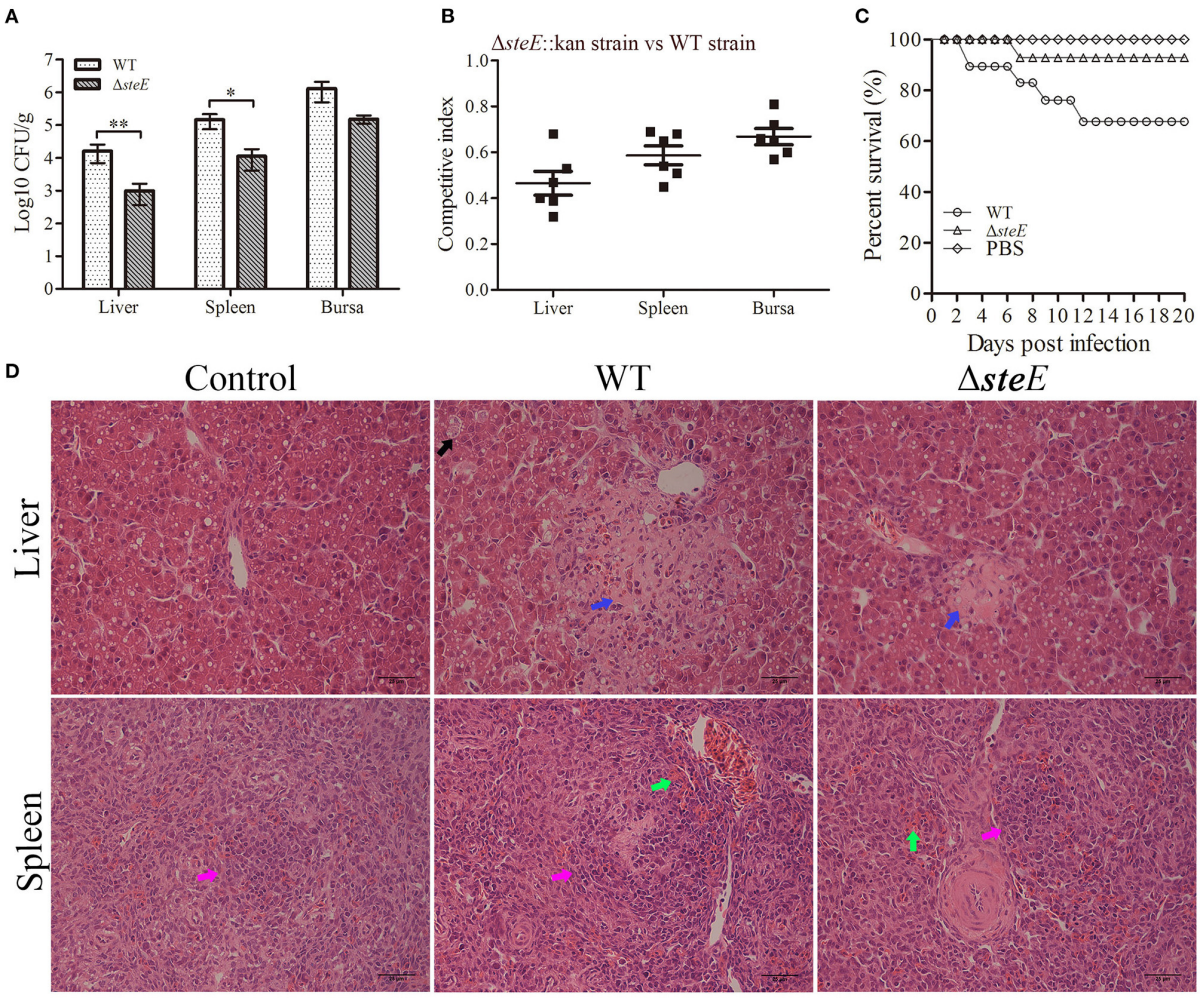
*S*. Pullorum Δ*steE* reduces virulence in chickens. **(A)** *steE* contributes to the colonization of the WT strain in chicken. Chickens were infected orally with 10^9^ CFUs of the WT and Δ*steE* strains. Bacterial recovery from the liver, spleen, and bursa was evaluated at 3 dpi. *p < 0.05; **p < 0.01. **(B)** *steE* contributes to WT strain growth in chickens. Chickens were infected orally with 2 × 10^8^ CFUs of a mixture of the Δ*steE*::Kan and WT strains prepared at a ratio of 1:1, and CFUs were evaluated from the liver, spleen, and bursa at 3 dpi using the plating assay. **(C)** *steE* contributes to WT virulence in chickens. The experimental groups were infected orally with 10^9^ CFUs of the WT or Δ*steE* strains, and the control group was administered PBS. The chickens were monitored daily for 20 dpi to evaluate the survival rate. **(D)** Histopathology of the liver and spleen tissues from chickens infected with the strains. Chickens were infected orally with 10^9^ CFUs of the strains, and the liver and spleen samples were collected at 3 dpi for histological analysis. Hematoxylin and eosin staining analysis showed the pathological changes in the infected livers and spleens (400 × ; scale bar: 25 μm).

To further investigate the effects of *steE* on colonization ability, we evaluated the bacterial load and long-term survival ability of the strains in chickens. Results showed that *steE* deletion decreased the colonization ability of *S*. Pullorum in the liver, spleen, and bursa of chickens at 3 dpi. The total number of bacteria recovered from the liver and spleen of the WT strain-infected chickens was significantly greater than those infected with Δ*steE* (Figure 7A). The competition index (CI) were <1, indicating that the deletion of *steE* reduced colonization, sustainability, and fitness of *S*. Pullorum relative to the WT strain in chickens (Figure 7B). Similar to the competitive index assays, the equivalent bacterial burden analysis showed that chicken inoculated orally with the Δ*steE* strain had a moderately long-term survival (Figure 7C). To examine the effect of *steE* deletion on the virulence of *S*. Pullorum, we compared the histopathological lesions induced by the WT and Δ*steE* strains in chickens. As shown in Figure 7D, the histopathological analysis showed a marked difference between chickens infected with the WT and Δ*steE* strains. The chickens infected with the Δ*steE* strain showed weak pathological lesions in the liver and spleen, including exudative nodules (black arrow), granular degeneration (blue arrow) of liver cells, splenic corpuscles (pink arrow) and congestion (green arrow). No significant damages were observed in the control group. Overall, the results from the chicken infection model suggest that *steE* is essential for virulence.

## Discussion

To date, more than 40 different effector proteins of the T3SSs encoded by SPI have been identified, which are involved in host-pathogen interaction. However, the effect of most effector proteins on the immune response of chicken macrophages and the pathogenicity of *S*. Pullorum is unclear (19). In this study, we showed that the effector protein SteE can enhance invasiveness and proliferation of *S*. Pullorum, which are required for bacterial survivability in host cells. Defensive responses can evolve in *Salmonella* to maintain a dynamic balance within the host, and these responses mediate the long-term survival and persistent infection of pathogens (20, 21). The findings of this study contribute to understanding the pathogenicity-related role of SteE during *S*. Pullorum infection, thus providing important clues for further studies.

The invasion and proliferation phenotypes of *Salmonella* are closely associated with bacterial virulence in macrophages (5). The T3SS2 is required for the intramacrophage replication of *S*. Typhimurium (22). Furthermore, the *steE* does not affect the invasion of *S*. Typhimurium in LCLs cells but result in its increased replication (7). In the present study, we found that *steE* increased the invasion and replication of *S*. Pullorum in HD-11 cells. Our results were not completely consistent with a previous report, with differences resulting due to the different cell lines used, MOI, or serotypes of *Salmonella* (7). Other intracellular bacteria, including *Mycobacterium tuberculosis, Brucella*, and *Francisella tularensis*, also affect the number of macrophages and promote bacterial survival during infection (23–25). Therefore, we hypothesized that the effector protein SteE, as a potential virulence factor, may contribute to invasiveness and proliferation.

Previous studies demonstrated that SPI-2 deletion significantly affected the expression of selective T3SS2 effector genes (6). In this study, deletion of *steE* significantly reduced the expression of selective T3SS virulence genes in *S*. Pullorum-infected HD-11 cells (Figure 3). We hypothesized that *steE* was closely related to the T3SS2-dependent virulence of *S*. Pullorum in HD-11 cells. Recent studies have evaluated the mechanisms underlying *steE*-mediated regulation of macrophage polarization and have provided insights into chronic infection with *Salmonella* (26, 27). Furthermore, SPI-2 effector mutants (*ssaV* and *steE*) in *S*. Typhimurium infected macrophages with significantly decreased IL-4 expression levels (28). Thus, *steE* may induce a competitive balance between the host microbiota and inflammation. Several studies have shown that *steE* can promote IL-10 production *via* the activation of STAT3 signaling and metabolic and physiological environment reprogramming for *Salmonella* in B cells, changing it from an anti-inflammatory to an infection state (7, 28, 29). In addition, the SteE effector protein increased the polarization of M2 macrophages in granulomas (11). Recently, Brodsky et al. obtained similar results that SteE protects the intracellular *Salmonella* from TNF-mediated granuloma clearance and hypothesized that SteE can polarize M2 macrophages (27). Furthermore, the *steE*-driven M2 granuloma macrophages polarization reduced iNOS mRNA expression compared to M1 granuloma macrophages, but the mRNA levels of IL-4 were significantly high (11, 29). The Δ*steE* strain induced a reduction in IL-10 levels in mice relative to *S*. Typhimurium (7). Our results confirmed that the effector *steE* promoted the production of anti-inflammatory cytokines (IL-4 and IL-10) in HD-11 and chicken spleens while reducing that of the pro-inflammatory cytokine iNOS in HD-11 cells (Figure 5), indicating that *steE* may provide a more permissive noninflammatory environment for *S*. Pullorum infection *in vivo* and *in vitro*.

The interaction between bacteriophages is also an important factor in bacterial virulence (Figure 1A). The functional prophages Gifsy-1 (*steE, gogB*, and *gipA*) and Gifsy-2 (*gtgE, sodCI*, and *gtgA*) of *Salmonella* affect the virulence of the pathogen and play a critical role in infecting host cells (8, 18, 30). *steE* is encoded within pathogenicity islands (Gifsy-1), which contributes to overcome host restriction during *Salmonella* infection (31). Our study confirmed that deletion of *steE* in *S*. Pullorum caused the increased virulence to Jinghong laying hens.GogB can interfere with NF-κB activation and reduce the host inflammatory response (10). GipA play an important role in replication of *S*. typhimurium within macrophages (17). The deletion of *gtgA* can significantly increase the virulence of *S*. Typhimurium in mice, indicating that a few effector proteins may play variable roles in different animal infection models that are conducive to *Salmonella* infection and cell survival (10). After animals are infected with *Salmonella*, the pathogen can spread through the epithelial cells or lymphoid tissues in the intestine. Infected phagocytes and free bacteria can translocate to the liver, spleen, and other organs and show aggregation and infiltration, resulting in systemic infection (31). Reportedly, *steE* significantly increased colonization of *S*. Typhimurium in mouse tissues (11, 32). In this study, deletion of *steE* attenuated the colonization of *S*. Pullorum to chickens. At the same time, the competition index and survival assays showed that the deletion of *steE* could reduce the fitness and persistence of *S*. Pullorum in chicken organs (Figure 7). Furthermore, *Salmonella*-containing vacuoles are formed in host cells, which provide a conducive environment for *Salmonella* proliferation, whereas *steE*-deficient *S*. Typhimurium reduced virulence in the BALB/c mice model (19, 33). From the results of our study, deletion of *steE* alleviated tissue injury and reduced the virulence of *S*. Pullorum in chickens, and these findings were consistent with *steE*-dependent regulation of inflammatory response *in vivo*. In future studies, we aim to include an analysis of the specific signaling pathways and mechanisms to improve our understanding of the interaction between the *S*. Pullorum effector SteE and host immune response.

To conclude, we demonstrated that *steE* was required for *S*. Pullorum invasion and proliferation and increased late apoptosis in HD-11 cells. Δ*steE* significantly reduced the expression of the anti-inflammatory cytokines IL-4 and IL-10 in infected HD-11 cells or chicken spleens, but significantly increased expression of IL-1β, IL-6 and IL-12 was detected compared to that in HD-11 cells and chicken spleens infected with *S*. Pullorum. Furthermore, deletion of *steE* significantly decreased colonization, pathological lesions, virulence, and long-term survival of *S*. Pullorum in the chicken infection model by regulating the inflammation response. Our results may provide interesting insights into the pathogenicity and immune response of *S*. Pullorum *steE* against the host.

## Data Availability Statement

The original contributions presented in the study are included in the article/Supplementary Material, further inquiries can be directed to the corresponding authors.

## Ethics Statement

The animal study was reviewed and approved by the Laboratory Animal Care and Ethics Committee of Henan Institute of Science and Technology (Permit Number: 2020HIST016).

## Author Contributions

ZL, TF, and JM conceived, designed the experiments, designed the research, and wrote the manuscript. ZL, YZ, and QW performed the experiments. YY and LW analyzed the data as well as interpretation of the data. ZL, PG, AF, and QW contributed analysis tools and revised the manuscript. All authors contributed to the article and approved the submitted version.

## Funding

This research was supported by National Natural Science Foundation of China-Henan Joint Fund (Grant Number U1904117) and Key Science and Technology Program of Henan Province (Grant Number 21210210100 and 212102110009).

## Conflict of Interest

The authors declare that the research was conducted in the absence of any commercial or financial relationships that could be construed as a potential conflict of interest.

## Publisher's Note

All claims expressed in this article are solely those of the authors and do not necessarily represent those of their affiliated organizations, or those of the publisher, the editors and the reviewers. Any product that may be evaluated in this article, or claim that may be made by its manufacturer, is not guaranteed or endorsed by the publisher.

## References

[1] Xu Z Qin Y Wang Y Li X Cao H Zheng SJ. A critical role of bacterioferritin in *Salmonella pullorum*-induced IFN-β expression in DF-1 cells. Front Microbiol. (2016) 7:20. 10.3389/fmicb.2016.00020 26870001 PMC4737897

[2] Tang Y Foster N Jones MA Barrow PA. Model of persistent *Salmonella* infection: *Salmonella enterica* serovar pullorum modulates the immune response of the chicken from a Th17-type response towards a Th2-type response. Infect Immun. (2018) 86:e00307–18. 10.1128/IAI.00307-18 29760212 PMC6056865

[3] Geng S Wang Y Xue Y Wang H Cai Y Zhang J. The SseL protein inhibits the intracellular NF-κB pathway to enhance the virulence of *Salmonella* pullorum in a chicken model. Microb Pathog. (2019) 129:1–6. 10.1016/j.micpath.2019.01.035 30703474

[4] Xian H Yuan Y Yin C Wang Z Ji R Chu C. The SPI-19 encoded T6SS is required for *Salmonella* pullorum survival within avian macrophages and initial colonization in chicken dependent on inhibition of host immune response. Vet Microbiol. (2020) 250:108867. 10.1016/j.vetmic.2020.108867 33010573

[5] Li Q Wang X Xia J Yuan Y Yin C Xu L. *Salmonella*-containing vacuole development in avian cells and characteristic of *cigR* in *Salmonella enterica serovar* pullorum replication within macrophages. Vet Microbiol. (2018) 223:65–71. 10.1016/j.vetmic.2018.07.013 30173754

[6] Yin J Xia J Tao M Xu L Li Q Geng S. Construction and characterization of a *cigR* deletion mutant of *Salmonella enterica serovar* pullorum. Avian Pathol. (2016) 45:569–75. 10.1080/03079457.2016.1187708 27163262

[7] Jaslow SL Gibbs KD Fricke WF Wang L Pittman KJ Mammel MK. *Salmonella* activation of STAT3 signaling by SarA effector promotes intracellular replication and production of IL-10. Cell Rep. (2018) 23:3525–36. 10.1016/j.celrep.2018.05.072 29924996 PMC6314477

[8] Pilar AVC Reid-Yu SA Cooper CA Mulder DT Coombes BK. GogB is an anti-inflammatory effector that limits tissue damage during *Salmonella* infection through interaction with human FBXO22 and Skp1. PLoS Pathog. (2012) 8:e1002773. 10.1371/journal.ppat.1002773 22761574 PMC3386239

[9] Yin C Liu Z Xian H Jiao Y Yuan Y Li Y. AvrA exerts inhibition of NF-κB pathway in its naïve *Salmonella* serotype through suppression of p-JNK and Beclin-1 molecules. Int J Mol Sci. (2020) 21:6063–76. 10.3390/ijms21176063 32842467 PMC7504150

[10] Takemura M Haneda T Idei H Miki T Okada N. A *Salmonella* type III effector, PipA, works in a different manner than the PipA family effectors GogA and GtgA. PLoS ONE. (2021) 16:e0248975. 10.1371/journal.pone.0248975 33735297 PMC7971870

[11] Pham THM Brewer SM Thurston T Massis LM Honeycutt J Lugo K. *Salmonella*-driven polarization of granuloma macrophages antagonizes TNF-mediated pathogen restriction during persistent infection. Cell Host Microbe. (2020) 27:54–67. 10.1016/j.chom.2019.11.011 31883922 PMC7065835

[12] Datsenko KA Wanner BL. One-step inactivation of chromosomal genes in *Escherichia coli* K-12 using PCR products. Proc Natl Acad Sci U S A. (2000) 97:6640–5. 10.1073/pnas.120163297 10829079 PMC18686

[13] Mu X Huan H Xu H Gao Q Xiong L Gao R. The transfer-messenger RNA-small protein B system plays a role in avian pathogenic *Escherichia coli* pathogenicity. J Bacteriol. (2013) 195:5064–71. 10.1128/JB.00628-13 24013628 PMC3811600

[14] Yin C Xu L Li Y Liu Z Gu D Li Q. Construction of pSPI12-cured *Salmonella enterica serovar* pullorum and identification of IpaJ as an immune response modulator. Avian Pathol. (2018) 47:410–7. 10.1080/03079457.2018.1471195 29712441

[15] Shi M Li N Xue Y Zhong Z Yang M. The 58th cysteine of TcpP is essential for *Vibrio cholera* virulence factor production and pathogenesis. Front Microbiol. (2020) 11:118. 10.3389/fmicb.2020.00118 32117142 PMC7017273

[16] Li Q Hu Y Chen J Liu Z Han J Sun L. Identification of *Salmonella enterica serovar* pullorum antigenic determinants expressed *in vivo*. Infect Immun. (2013) 81:3119–27. 10.1128/IAI.00145-13 23774596 PMC3754199

[17] Klumpp J Fuchs TM. Identification of novel genes in genomic islands that contribute to *Salmonella* Typhimurium replication in macrophages. Microbiology. (2007) 153:1207–20. 10.1099/mic.0.2006/004747-0 17379730

[18] Coombes BK Wickham ME Brown NF Lemire S Bossi L Hsiao WW. Genetic and molecular analysis of GogB, a phage-encoded type III-secreted substrate in *Salmonella enterica serovar* Typhimurium with autonomous expression from its associated phage. J Mol Biol. (2005) 348:817–30. 10.1016/j.jmb.2005.03.024 15843015

[19] Johnson R Mylona E Frankel G. Typhoidal *Salmonella*: distinctive virulence factors and pathogenesis. Cell Microbiol. (2018) 20:e12939. 10.1111/cmi.12939 30030897

[20] Lawley TD Chan K Thompson LJ Kim CC Govoni GR Monack DM. Genome-wide screen for *Salmonella* genes required for long-term systemic infection of the mouse. PLoS Pathog. (2006) 2:0087–100. 10.1371/journal.ppat.0020011 16518469 PMC1383486

[21] Hannemann S Gao B Galán JE. *Salmonella* modulation of host cell gene expression promotes its intracellular growth. PLoS Pathog. (2013) 9:e1003668. 10.1371/journal.ppat.1003668 24098123 PMC3789771

[22] Figueira R Watson KG Holden DW Helaine S. Identification of *Salmonella* pathogenicity island-2 type III secretion system effectors involved in intramacrophage replication of *S.* enterica serovar Typhimurium: implications for rational vaccine design. mBio. (2013) 4:e00065. 10.1128/mBio.00065-13 23592259 PMC3634603

[23] Kerrinnes T Winter MG Young BM Diaz-Ochoa VE Winter SE Tsolis RM. Utilization of host polyamines in alternatively activated macrophages promotes chronic infection by brucella abortus. Infect Immun. (2018) 86:e00458–17. 10.1128/IAI.00458-17 29203548 PMC5820950

[24] Refai A Gritli S Barbouche MR Essafi M. *Mycobacterium tuberculosis* virulent factor ESAT-6 drives macrophage differentiation toward the pro-inflammatory M1 phenotype and subsequently switches it to the anti-inflammatory M2 phenotype. Front Cell Infect Microbiol. (2018) 8:327–40. 10.3389/fcimb.2018.00327 30283745 PMC6157333

[25] Jiang L Wang P Song X Zhang H Ma S Wang J. *Salmonella* Typhimurium reprograms macrophage metabolism via T3SS effector SopE2 to promote intracellular replication and virulence. Nat Commun. (2021) 12:879. 10.1038/s41467-021-21186-4 33563986 PMC7873081

[26] Eisele NA Ruby T Jacobson A Manzanillo PS Cox JS Lam L. *Salmonella* require the fatty acid regulator PPARδ for the establishment of a metabolic environment essential for long-term persistence. Cell Host Microbe. (2013) 14:171–82. 10.1016/j.chom.2013.07.010 23954156 PMC3785333

[27] Brodsky IE. JAK-ing into M1/M2 polarization SteErs *Salmonella*-containing macrophages away from immune attack to promote bacterial persistence. Cell Host Microbe. (2020) 27:3–5. 10.1016/j.chom.2019.12.007 31951822

[28] Stapels DAC Hill PWS Westermann AJ Fisher RA Thurston TL Saliba AE. *Salmonella* persisters undermine host immune defenses during antibiotic treatment. Science. (2018) 362:1156–60. 10.1126/science.aat7148 30523110

[29] Panagi I Jennings E Zeng J Günster RA Stones CD Mak H. *Salmonella* effector SteE converts the mammalian serine/threonine kinase GSK3 into a tyrosine kinase to direct macrophage polarization. Cell Host Microbe. (2020) 27:41–53. 10.1016/j.chom.2019.11.002 31862381 PMC6953433

[30] Ho TD Figueroa-Bossi N Wang M Uzzau S Bossi L Slauch JM. Identification of GtgE, a novel virulence factor encoded on the Gifsy-2 bacteriophage of *Salmonella enterica* serovar Typhimurium. J Bacteriol. (2002) 184:5234–9. 10.1128/JB.184.19.5234-5239.2002 12218008 PMC135366

[31] Neves P Lampropoulou V Calderon-Gomez E Roch T Stervbo U Shen P. Signaling via the MyD88 adaptor protein in B cells suppresses protective immunity during *Salmonella* Typhimurium infection. Immunity. (2010) 33:777–90. 10.1016/j.immuni.2010.10.016 21093317

[32] Niemann GS Brown RN Gustin JK Stufkens A Shaikh-Kidwai AS Li J. Discovery of novel secreted virulence factors from *Salmonella enterica serovar* Typhimurium by proteomic analysis of culture supernatants. Infect Immun. (2011) 79:33–43. 10.1128/IAI.00771-10 20974834 PMC3019877

[33] Stévenin V Chang YY Toquin LY Duchateau M Gianetto QG Luk CH. Dynamic growth and shrinkage of the *Salmonella*-containing vacuole determines the intracellular pathogen niche. Cell Rep. (2019) 29:3958–73. 10.1016/j.celrep.2019.11.049 31851926 PMC6931108

