# Supplementary figures and images for: SteE Enhances the Virulence of Salmonella Pullorum in Chickens by Regulating the Inflammation Response
Source: Front Vet Sci. 2022 Jul 14;9:926505. doi: 10.3389/fvets.2022.926505 (PMC9330158; doi:10.3389/fvets.2022.926505)

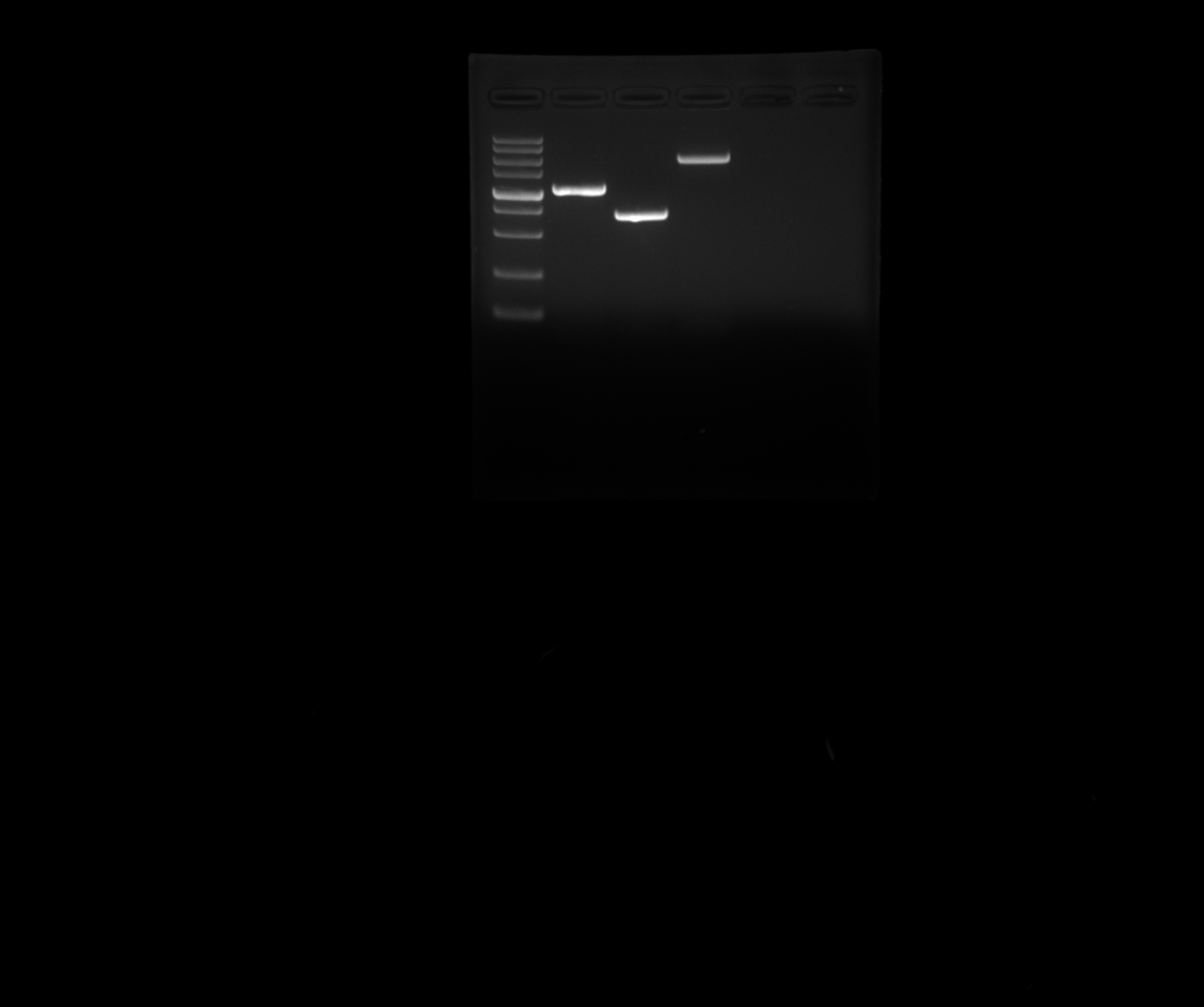

Supplement: Supplementary file 1 [file Data_Sheet_1.ZIP › Supplementary Images/Figure 1B.Tif]

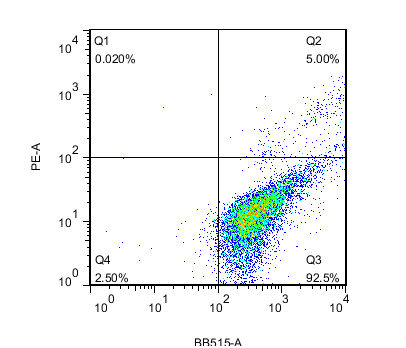

Supplement: Supplementary file 1 [file Data_Sheet_1.ZIP › Supplementary Images/Figure 4-WT.PNG]

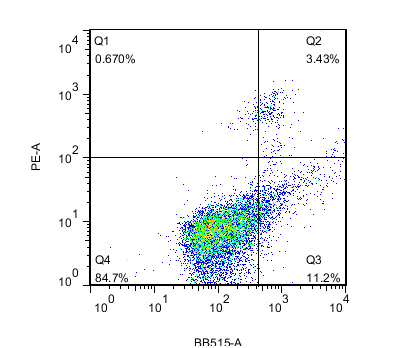

Supplement: Supplementary file 1 [file Data_Sheet_1.ZIP › Supplementary Images/Figure 4-control.PNG]

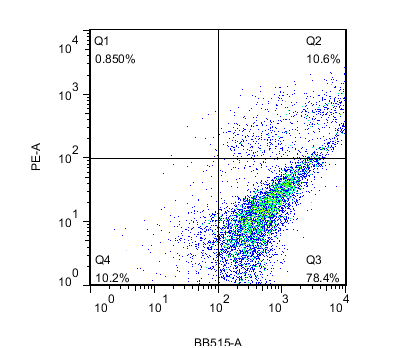

Supplement: Supplementary file 1 [file Data_Sheet_1.ZIP › Supplementary Images/Figure 4-añsteE.PNG]

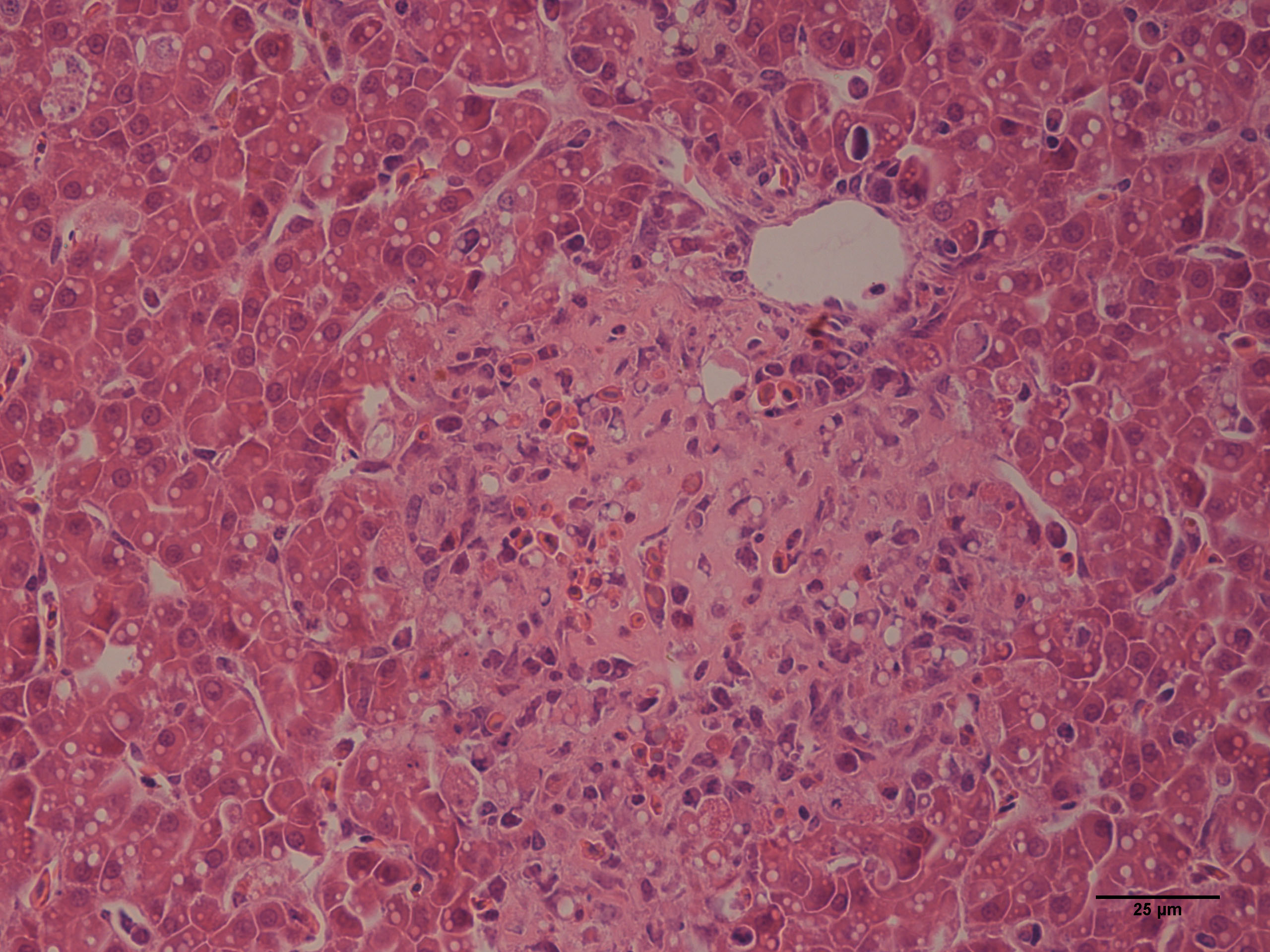

Supplement: Supplementary file 1 [file Data_Sheet_1.ZIP › Supplementary Images/Figure 7D-WT-liver.jpg]

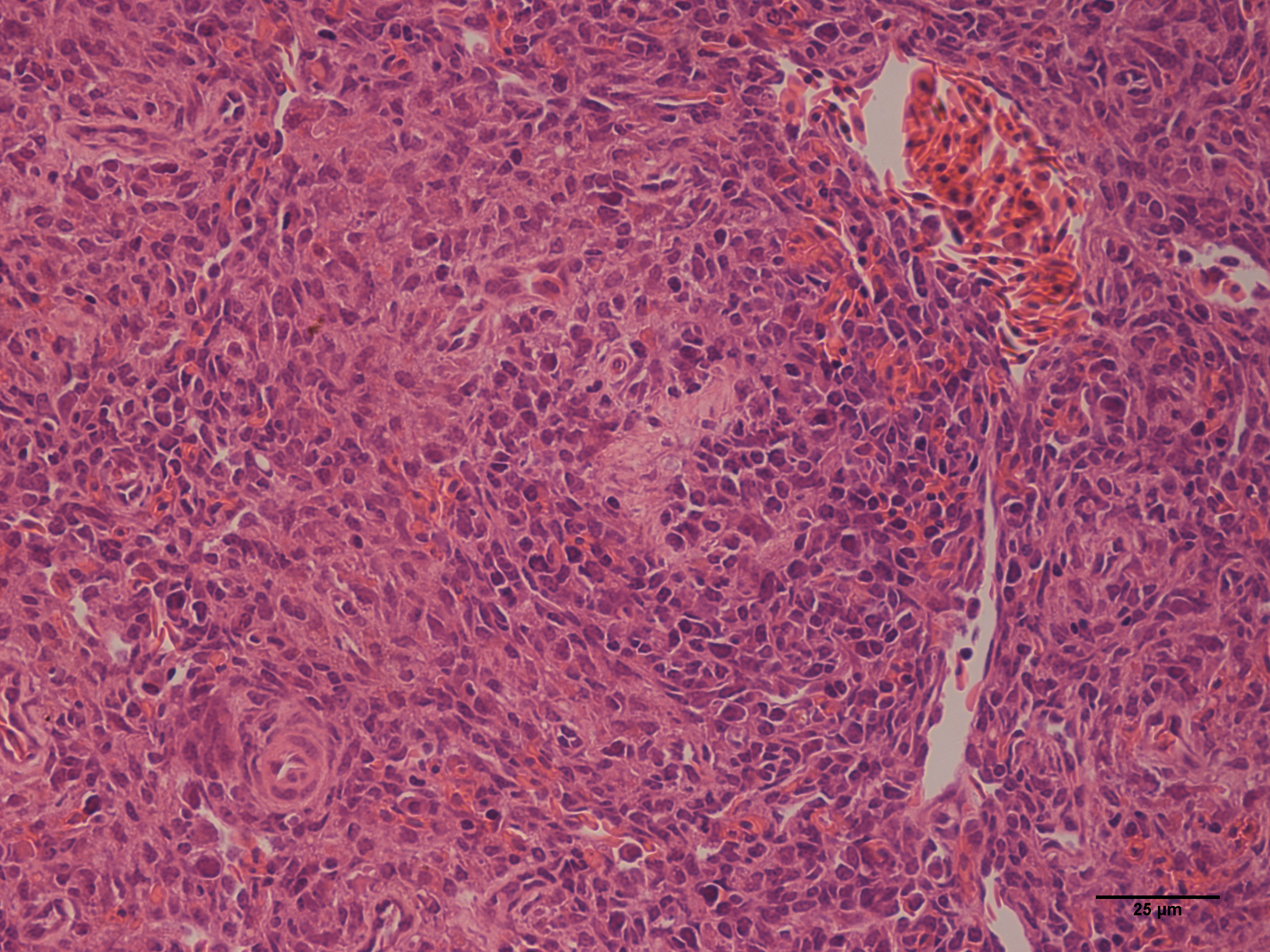

Supplement: Supplementary file 1 [file Data_Sheet_1.ZIP › Supplementary Images/Figure 7D-WT-speen.jpg]

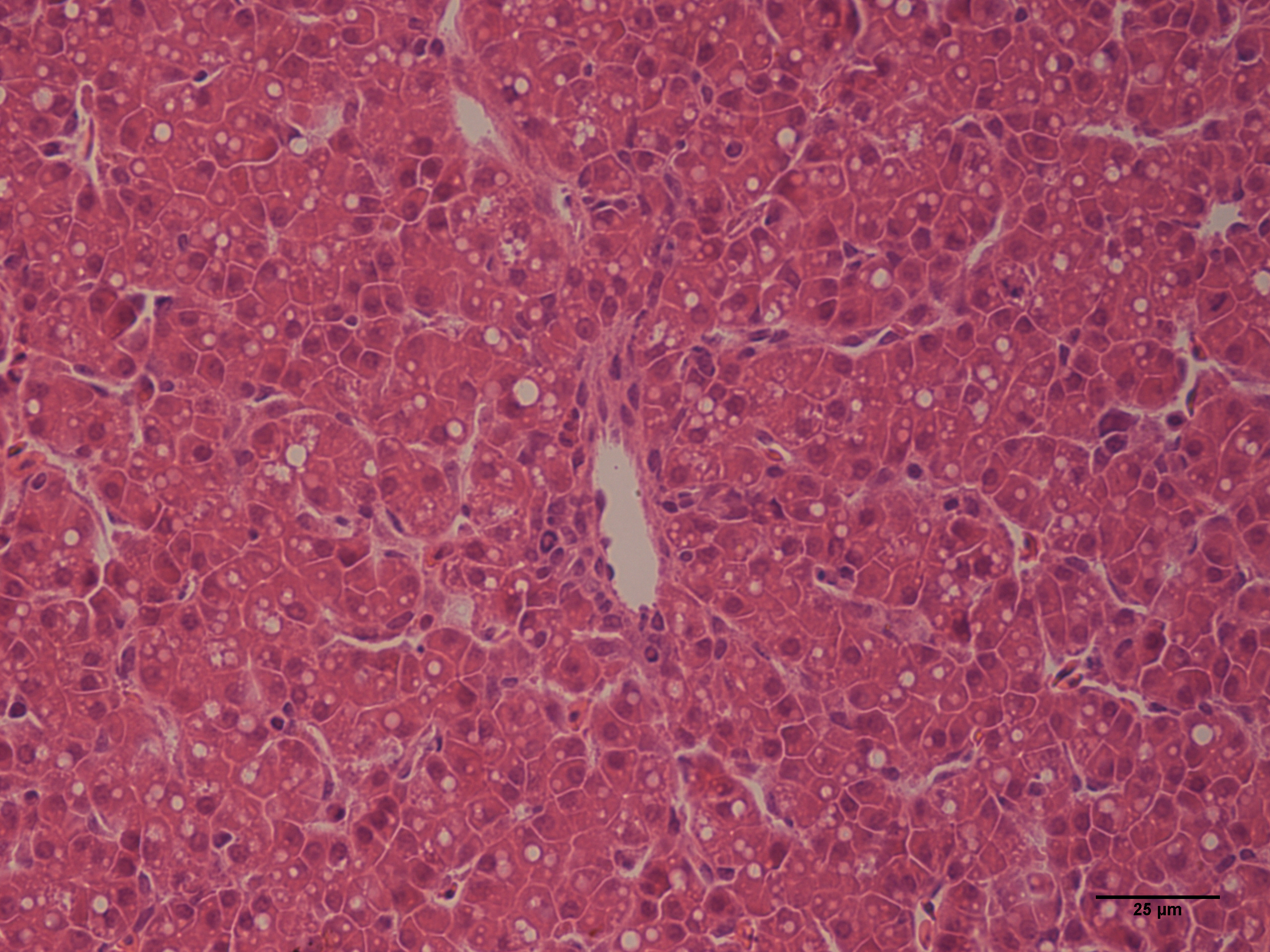

Supplement: Supplementary file 1 [file Data_Sheet_1.ZIP › Supplementary Images/Figure 7D-control-liver.jpg]

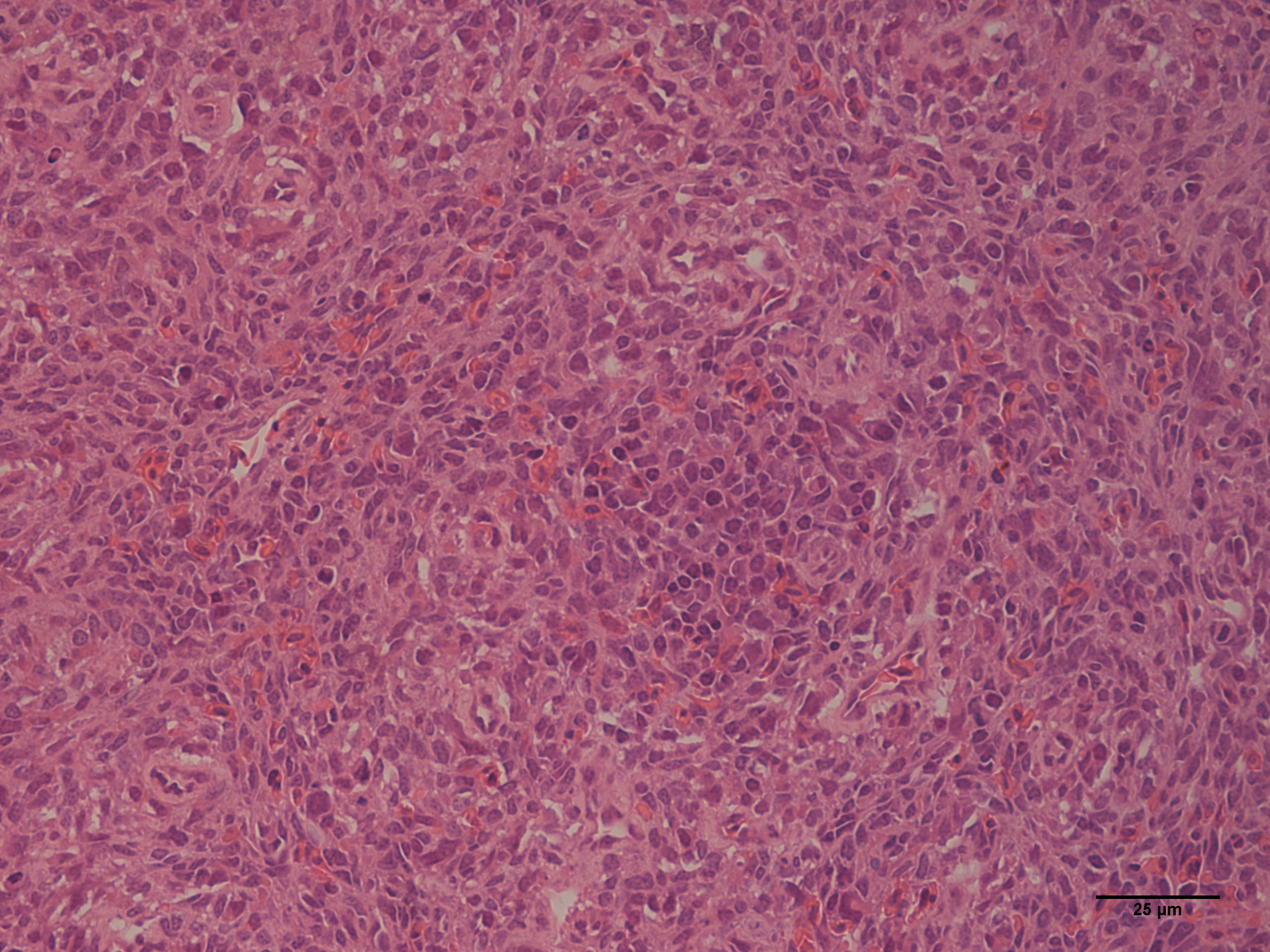

Supplement: Supplementary file 1 [file Data_Sheet_1.ZIP › Supplementary Images/Figure 7D-control-speen.jpg]

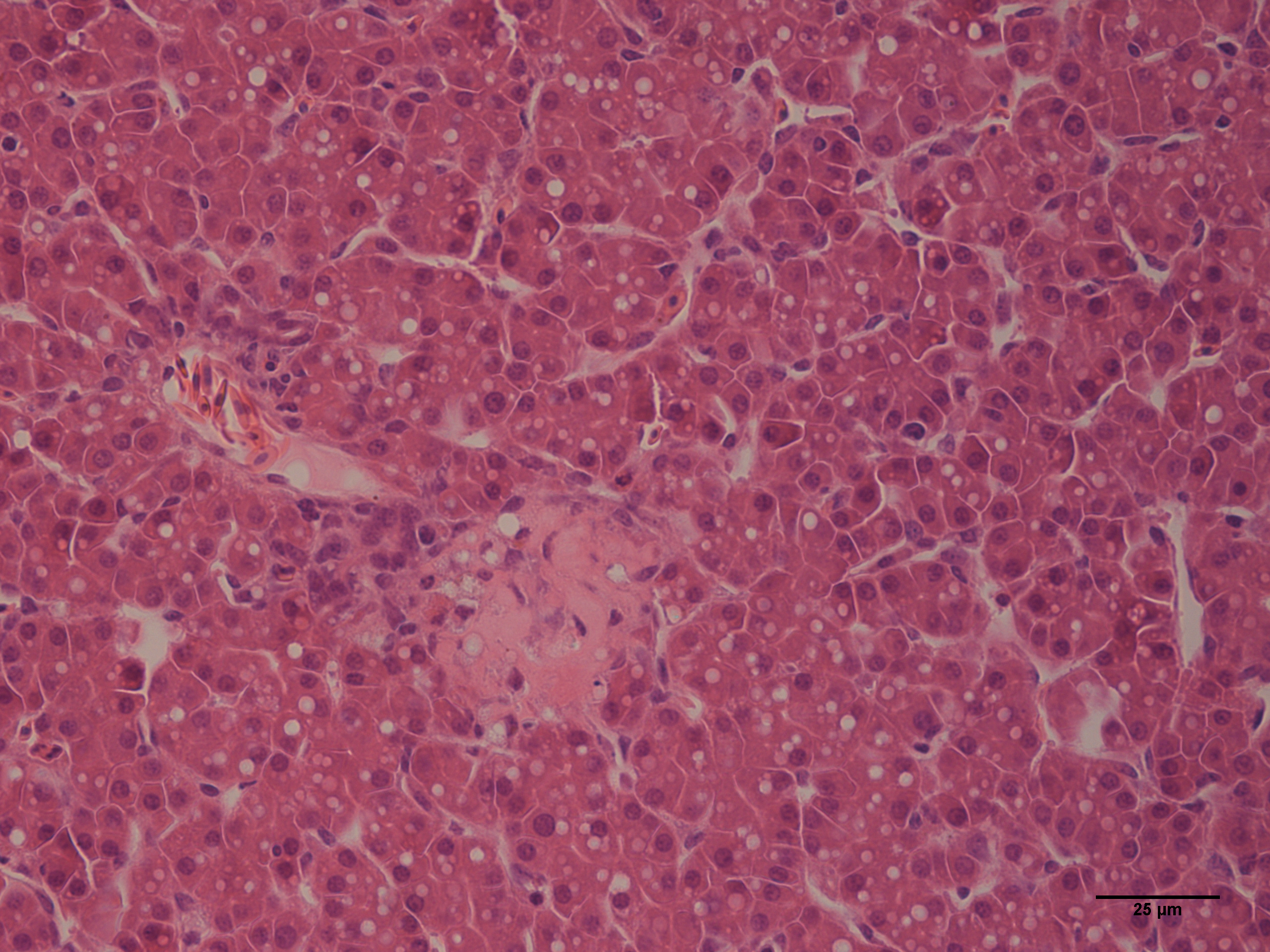

Supplement: Supplementary file 1 [file Data_Sheet_1.ZIP › Supplementary Images/Figure 7D-añsteE-liver.jpg]

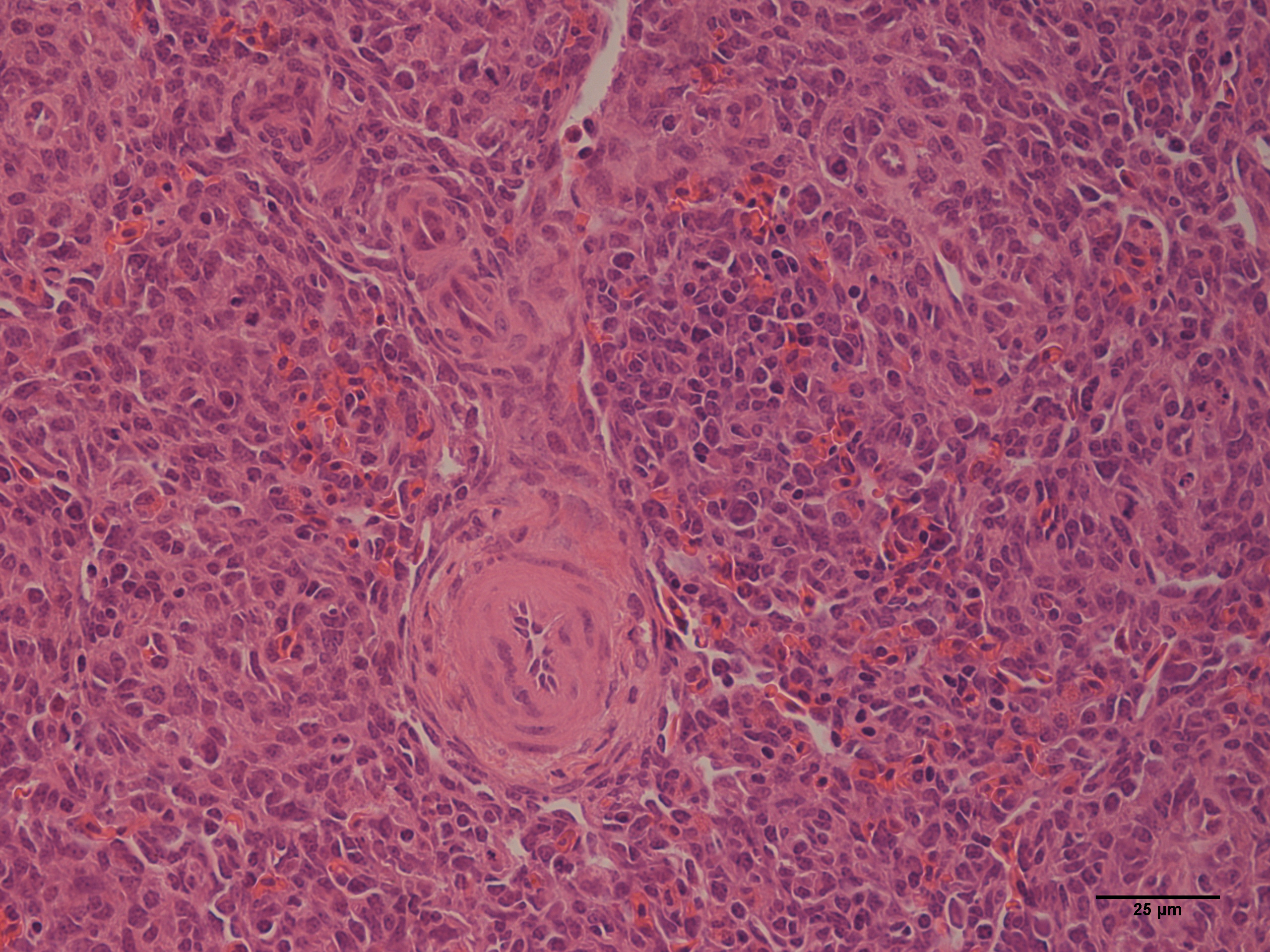

Supplement: Supplementary file 1 [file Data_Sheet_1.ZIP › Supplementary Images/Figure 7D-añsteE-speen.jpg]
